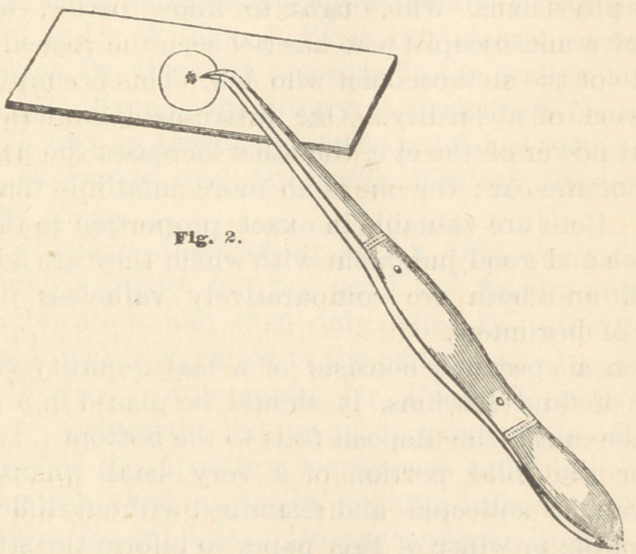# The Microscope in Daily Practice, (Third Paper)

**Published:** 1875-11

**Authors:** I. N. Danforth

**Affiliations:** Lecturer on Pathology in Rush Medical College, Chicago; 74 South Morgan Street


### THE MICROSCOPE IN DAILY PRACTICE.

(THIRD PAPER.)

BY I. N. DANFORTH, M.D.,

LECTURER ON PATHOLOGY IN RUSH MEDICAL COLLEGE, CHICAGO.

It is one thing to *buy* a microscope ; it is quite another thing to make it practically valuable in the practice of medicine. There are two classes of extremists with respect to the microscope ; one class ridicule it as of no value, the other repose in it a profound faith and regard it as well nigh infallible. These two classes are about equi-distant from the truth, but of the two I prefer the former, since it is generally easier to remove a reasonable and *reasoning* skepticism than to curb an unreasoning and unreasonable faith—and, I may add, far more satisfactory. To the physician in every day practice, the microscope is mainly useful as an aid to diagnosis ; in this respect, it takes its place with the ophthalmoscope, the stethoscope, the laryngoscope, the otoscope, and the rest of the "scopes" that are or are to be. But the microscope is, or ought to be, more than this ; as Stricker tersely puts it, it "is a means of research," and every physician who owns a microscope should so regard it and so *use* it. The young men of our profession in the Northwest are as capable of original research as are the young men of England, France or Germany. It is not to our credit that we are compelled to go abroad for most of the new discoveries in physiology and pathology. Americans make the best microscopes and the fewest discoveries in medicine ; Germany the poorest microscopes and the most discoveries. How natural it is for us to hang our faith in matters relating to histology upon the Germans ; but in doing this we export all the compliments.

It is not very difficult, however, to understand why so many of our students and graduates let the microscope severely alone. I am persuaded that it is largely due to the fact that microscopy is commonly looked upon as a very mysterious as well as a very expensive piece of business. It seems to be generally believed that a large array of costly instruments and apparatus is necessary, in addition to a costly microscope—so that it is by no means singular that the student or physician of moderate means should look upon the microscope as something *desirable* but not *attainable.*

In the two preceding issues of the JOURNAL AND EXAMINER I tried to show that a good microscope need not be a costly affair; in this present paper I propose to show that it does not demand a very great outlay of *money* to *get ready* to use the instrument.

The specimens, both healthy and morbid, which will present themselves to the physician for examination may be divided, for convenience, into three classes:

(*a*) Fluids.

(*b*) Semi-solid structures.

(*c*) Solid structures.

For the examination of all sorts of specimens, slips of glass of convenient size will be required. These glass slips or slides are now universally made three inches long by one inch wide, and they can be purchased of any dealer in "microscope findings" at about fifty cents per dozen. A uniform size has been adopted so that *all* slides will fit securely in *all* cases or cabinets; it is of course merely a matter of general convenience; we all agree to uniformity in this matter just as we all agree to a uniform size of screw for objectives and other accessories. It is not, however, necessary to buy the ground slides. A thin pane of common window glass, selected with some care, may be cut into slides, and will answer every purpose, with the exception of now and then a slide that will contain "flaws," like air bubbles or scratches or other irregularities. But these slides made of window glass, will answer every practical purpose. "Covering" glasses will likewise be required from the outstart. These are round or square pieces of very thin glass prepared especially for the purpose of covering the object, after it has been placed upon the glass slide, the design being to flatten the specimen, and thus cause it to assume a uniform degree of thickness. These thin circles or squares of glass are kept by the dealers, and they generally cost about forty cents per dozen for common sizes, as five-eighths or three-quarters of an inch diameter or square, as the case may be. But here, again, for ordinary use, a substitute may be employed which is vastly cheaper and far more durable. Let the student procure a piece of clear mica (from any stove dealer) and carefully split it into thin laminæ; then let each lamina be cut into squares or circles as may be desired. The objection to mica is, that it is less clear than glass, and that it is likely to show striæ or scratches, corresponding with the various laminations. But the student will shortly become accustomed to these defects, so that he will scarcely notice them at all; in other words, they practically cease to be defects, just as blemishes of speech or feature cease to be repugnant as we acquire familiarity with them.

(*a*) *The Examination of Fluids.* The microscopic examination of the fluid substances which come under the notice of the physician, really means the study of their solid portions, which are sometimes amorphous, sometimes crystalline, sometimes cellular. The mode of procedure is very simple, and is essentially the same for all fluid products, if the specimen consists of as much as a couple of fluid drachms. Pour the specimen into a conical glass, like an old fashioned wine glass, cover it carefully to prevent the access of dust, and allow it to remain until the sediment falls to the bottom, which will take from six to twenty-four hours, according to the copiousness and density of the sediment.

For the purpose of gathering the sediments of urine, serous discharges, etc., conical glasses of four or five ounces capacity are sold by the dealers in microscope findings and chemical wares; they are very convenient, but not *necessary*. An old fashioned wine glass, which can be found in almost any house, or country store, answers equally well, and costs far less; in the absence of this, a test tube will answer almost as well, and as a matter of absolute fact, in nine times out of ten, a sufficient quantity of the sediment can be obtained, if the specimen is merely allowed to stand in an ordinary druggist's phial.

After precipitation has taken place, the next thing is to get a drop of the precipitate in shape for examination. For the removal of a minute quantity of the deposit from the bottom of the conical glass, or other vessel, a *pipette* is necessary. This is merely a glass tube, about three-sixteenths of an inch in external diameter, and ten or twelve inches long. These tubes are generally drawn to a capillary point, by simply heating them in the flame of an alcohol lamp—an operation which the merest tyro can easily perform. Then take the tube between the thumb and second finger, and close the upper orifice with the index finger, so as to prevent the escape of air; plunge the lower end of the pipette to the bottom of the glass containing the specimen, and slightly raise the index finger from the upper orifice; a few drops of the sediment will flow into the tube, which should then be quickly closed again with the finger; now raise the tube, still tightly closed, from the glass, wipe the adhering fluid from its external surface, and allow a single drop of the contained sediment to flow upon a glass slide, previously prepared for its reception. If the student does not happen to possess a pipette, a rye or wheat straw, or the stem of a common clay pipe can be made to answer almost as well. "Where there is a will there is a way"—a bit of philosophy which, I am bound to confess, is not exactly original with me.

Having placed the specimen upon the glass slide, the next step is to apply the thin glass or mica "cover"—and it requires some little tact to do this, without including a colony of pestering air bubbles.

For the application of the covering glass, as well as for many other purposes in connection with microscopy, a small sharp-pointed, curved forceps, like that represented in the accompanying illustration (Fig. 1), will be

Fig. 1.

found extremely useful. They can be obtained for about 50 cts. each of Messrs. Johnson & Lund, dealers in dental supplies, of this city. Those with comparatively weak springs should be selected, as it is very tiresome to the fingers to hold the points in firm contact if the resistance is too great. The glass cover is applied in the following

Fig. 2.

manner: Take the forceps as represented in the illustration (Fig. 2), and, with the curved points of the instrument, seize the "cover" near its margin, place the opposite (or free) margin in contact with the slide, and allow the cover to descend slowly and gently upon the specimen to be examined, until it is nearly in contact with the slide; then allow it to fall of its own weight. The specimen will be included between the slide and the cover, and the effect of the latter will be to spread it out in a thin film of uniform thickness. As the covering-glass falls, a portion of the fluid will be forced out around the margins; this must be absorbed by a fragment of blotting paper, or a bit of old muslin. The specimen is now ready for examination, and the student should proceed to study it, slowly, carefully, and *thoughtfully.* I particularly insist upon the importance of comparing the microscopic appearances of the morbid specimen with the history of the case. In renal diseases this is *imperative*, if the diagnosis and prognosis are to be of any *certain* value. No thoughtful, careful physician trusts to his stethoscope, or his ophthalmoscope, or his laryngoscope *alone;* no more can he trust to his microscope *alone.* Many physicians, who ought to know better, expect more of a microscopist who has *not* seen the patient than they do of the stethoscopist who *has.* This is simply the perfection of absurdity. One instrument increases the natural power of the eye, the other increases the natural power of the ear; the one is no more infallible than the other. Both are valuable in exact proportion to the experience and good judgment with which they are administered, and both are comparatively valueless in the hands of beginners.

When a specimen consists of a less quantity than a couple of fluid-drachms, it should be placed in a small test tube until the deposit falls to the bottom. In this manner the solid portion of a very small quantity of fluid can be collected and examined without difficulty. It is not the province of this paper to inform the student *what* he is liable to find in the many varieties of fluid specimens which may come before him, nor the pathological import of the microscopic appearances. These are matters that will be taken up in their appropriate places, later in the series.

74 South Morgan Street.

## Figures and Tables

**Fig. 1. f1:**
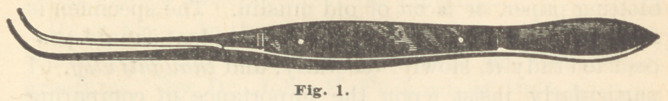


**Fig. 2. f2:**